# Combinational inhibition of EGFR and YAP reverses 5-Fu resistance in colorectal cancer

**DOI:** 10.7150/jca.44775

**Published:** 2020-07-11

**Authors:** Changhao Huang, Zihua Chen, Chen Yang, Lu Chen, Chen Lai, Yingying Zhang, Weijie Yuan, Ji-Hak Jeong

**Affiliations:** 1 Department of Gastrointestinal Surgery, Xiangya Hospital, Central South University, Changsha, Hunan, 410008, China; 2 Department of Oncology, Xiangya Hospital, Central South University, Changsha, Hunan, 410008, China; 3 Research Institute of Pharmaceutical Sciences, College of Pharmacy, Kyungpook National University, 80 Daehak-ro, Buk-gu, 41566, Daegu, Republic of Korea

**Keywords:** colorectal cancer, YAP, chemotherapy resistance, EGFR, 5-Fu

## Abstract

Yes-associated protein (YAP) is a transcriptional coactivator that promotes cell proliferation, migration, and tissue homeostasis in colorectal cancer (CRC). Here, we established 5-Fu resistant CRC cell line (SW620R) and examined the role of YAP in chemotherapy resistance. We showed that YAP promoted cell proliferation, migration, and chemotherapy resistance in CRC. To increase efficacy of CRC treatment, we employed another therapeutic target EGFR which interacts with the upstream signaling molecules of YAP in Hippo pathway. Verteporfin, a YAP specific inhibitor, inhibits YAP activity by blocking the YAP-TEAD complex in the cell nucleus, and AG1478, an inhibitor of EGFR/ErbB1, induces the phosphorylation and degradation of YAP. We found that combinational inhibition of YAP by VP and AG1478 synergistically suppressed the CRC development and reversed chemotherapy resistance *in vitro* and *in vivo*. Therefore, our results demonstrated a novel therapeutic strategy, the combination of inhibitors targeting EGFR and YAP, to suppress and reverse chemotherapy resistance in colorectal cancer.

## Introduction

Colorectal cancer (CRC) is the third most common malignant tumor (for incidence and mortality) in men and second in women in the world 1. In 2018, over 1.8 million new colorectal cancer cases and 881,000 deaths are estimated to occur 2. CRC patients in their progressive stage could often benefit from the neoadjuvant chemotherapy based on combination of Fluorouracil (5-Fu) and platinum-based drugs in the colorectal radical surgery period. More than 80% advanced CRC patients eventually develop relapsed disease despite their initial response to chemotherapy 3. Thus, the 5-Fu resistance is the key barrier of improving therapy efficacy in CRC patients.

The Hippo pathway, highly conserved in mammalian, regulates intrinsic organ sizes by regulating apoptosis and cell proliferation. Yes-associated protein (YAP) is a transcriptional effector of the Hippo pathway 4. It is the key event of Hippo pathway for mediating cancer cell proliferation and migration that YAP is play a role by being phosphorylated and translocated into the cell nucleus 5, 6. YAP is maintained as highly active form in human malignancies, which suggests that YAP can be an attractive therapeutic target of cancer treatment. Verteporfin (VP), a benzoporphyrin derivative, is clinically used as a photosensitizer and recently known to suppress the Hippo pathway signaling by blocking the interaction between YAP and TEA domain transcription factor 7. Therefore, the conventional chemotherapeutics combined with VP could potentiate the chemotherapy effectiveness, overcoming its acquired resistance to initial chemotherapy 8.

EGFR tyrosine kinase inhibitors (EGFR-TKIs), such as cetuximab, gefitinib, AG1478, and erlotinib, have been commonly used in metastatic gastrointestinal cancer and have been proved to improve progression-free survival 9, 10. In colorectal cancer cells, VP can reverse primary resistance to EGFR inhibition 11. Since dual inhibition targeting EGFR and YAP could provide better therapeutic effect than single inhibition of each 12, we set out to explore if the combination of YAP and EGFR inhibition reverses chemotherapy resistance in colorectal cancer.

In the present study, we investigated the effect of combinational inhibition of YAP and EGFR on 5-Fu resistance in CRC. We showed that 5-Fu resistance upregulated YAP protein levels in 5-Fu resistant CRC cells, which could be a prognosis marker for 5-FU-based treatment. In addition, we found that combinational inhibition of YAP and EGFR reversed 5-Fu resistance in CRC *in vivo* and *in vitro*. Our study provided the underlying mechanisms for 5-Fu resistance in CRC and therapeutic strategy of combinational therapy for reversing the chemotherapy resistance in CRC.

## Materials and Methods

### Patient selection and tissue microarray preparation

Patients enrolled into the current study met the following criteria: 1. Patients were admitted to Department of Gastrointestinal Surgery of Xiangya Hospital of Central South University (Changsha, China) from July 2017 to December 2018. 2. Patients were diagnosed by pathological review of tumor biopsies along with enhanced abdominal CT or MRI scan. 3. Radical resection of colorectal cancer was conducted to the patients. 4. Post-operative adjuvant chemotherapy based on fluorouracil (5-FU) were performed for 6-12 cycles in six months. Patients who received neoadjuvant therapy before surgery were excluded. 84 patients were called each month and followed up at regular intervals. The cancer relapse of these patients was diagnosed by increasing the serum tumor markers and imagological examination. The study was approved by the Research Ethics Committee of Xiangya Hospital, Central South University.

Cancer tissues were excised and fixed in 10% neutral-buffered formalin and then embedded in paraffin blocks. Each paraffin-embedded section was cut 4 μm thick, deparaffinized, and rehydrated. HE staining was performed to detect and mark typical gastric adenocarcinoma sections in CRC tissues, and the obvious normal gastric mucosa in CRC adjacent tissues was evaluated by a professional pathologist.

### Immunohistochemistry

Immunohistochemical staining for YAP (1:200), and EGFR (1:400) was performed on the tissue slides. Negative controls were prepared by substituting the primary antibody with non-immune goat serum. 4 areas on each slide were randomly chosen for IHC scoring. The staining results were evaluated by two independent pathologists (double-blinded) at the same time. Means were taken for final analysis. The samples in which the staining intensity was none or weak and less than half cells were stained were rendered negative (-), while the samples with moderate or strong staining in more than half cells were positive (+).

### Cells and Reagents

SW620, Colo205, HCT15, and HCT116 cells were cultured in RPMI 1640 medium supplemented with 1% antibiotic-antimycotic and 10% fetal bovine serum (FBS) and incubated at 37°C in a humidified atmosphere containing 5% CO_2_. VP (verteporfin), 5-Fu (5-Fluoracil), and AG-1478 were purchased from Selleckchem (USA).

### Establishing 5-Fu resistant cell line

Four-week-old male athymic NOD/SCID mice were used to establish chemotherapy-resistance model. The mice were subcutaneously injected with SW620 cells (2×10^6^ cells in 200 μL volume). After 14 days of inoculation, 5-FU by IP injection at 30mg/kg/mouse was applied thrice a week for four weeks. After four weeks, the mice were sacrificed and tumors were collected and digested into primary cells, SW620R. To obtain 5-Fu resistant cell line, tumor cells were isolated and purified by using ACCUMAX^TM^ (Innovative Cell Technologies) according to manufacturer's instructions. In brief, the tumor tissues were rinsed with sterile DPBS twice, and transferred to a petri dish containing sterile DPBS. The tissues were cut by surgical scissors into small pieces approximately 1 mm in size, then transferred to 50 ml sterile centrifuge tube. The pieces were settled by centrifugation and carefully removed the supernatant for two times. The pieces were transferred to new 50 ml sterile centrifuge tube and treated with ACCUMAX^TM^, and then incubated on an agitator at RT for 30 minutes. After the incubation, the cells were isolated by cell strainers, then removed the supernatant by centrifugation at 900 rpm for 5 minutes. The cells were resuspended in DPBS and centrifuged for washing two times. After washing, the primary cells (SW620R) were cultured in RPMI1640 medium containing 5-FU (concentration 1 μM).

### Xenograft tumor model

The four-week-old male mice were subcutaneously injected with SW620R (2×10^6^ cells in 200 μL volume). After 14 days of inoculation, the mice were treated with either vehicle (control), 5-FU (30mg/kg*3/week, ip), or the combination of VP, AG1478, and 5-FU (VP 50mg/kg*3/week, AG1478 50mg/kg*3/ week, 5-FU 30mg/kg*3/ week, ip) for 2 weeks. Then, the mice were sacrificed, and the tumors were collected and weighted.

### Cell proliferation assay

The cells were incubated in 96-well plates at a density of 4×10^3^ cells per well overnight. At different points, 10 μL of MTT dye was added and incubated for 4 hr at 37 °C. Then, the original media was removed, and 100 μL of DMSO was added to each well and shaken for 10 min. The spectrometric absorbance at the wavelengths of 570 and 630 nm was determined with a microplate reader (Tecan, USA).

### Western blot analysis

Anti-YAP antibody (#14074) and anti-EGFR antibody (#4267) were purchased from Cell Signaling Technology (Beverly MA, USA). The transferred membranes were subsequently incubated overnight (more than 16 hr) at 4 °C with the primary antibody (1:1000) and then the secondary antibody (1:3000) for 1 hr. Chemoluminescence detection was performed by using the Pierce ECL Western Blotting Substrate (Thermo Scientific).

### Transduction

When SW620 cells reached 80%-90% confluence on the day of transduction, the three kinds of lentiviral stock (YAP-shRNA, EGFR-shRNA, and Control-shRNA, purchased from Sigma) were respectively transduced into the cells with PEI (Polyethylenimine). After 24 hr, these cells were replated in culture plates to obtain temporary transduction cells.

### Colony formation assay

A total of 500 stable-transfected CRC cells were seeded into each well of a six-well plate and incubated with 10% FBS media for 15 days, with the media replaced every 3-5 days. After 15 days, the colonies were fixed with formalin and stained with 0.1% crystal violet (Sigma Aldrich).

### Wound healing assay

The monolayer of the cells was wounded by dragging a 10 μL pipette tip. The cells were washed to remove cellular debris and then migrated for 12 hr. Images were captured under an inverted microscope.

## Results

### The expression of YAP and EGFR is manifested in recurrent human CRC

**Figure 1:**
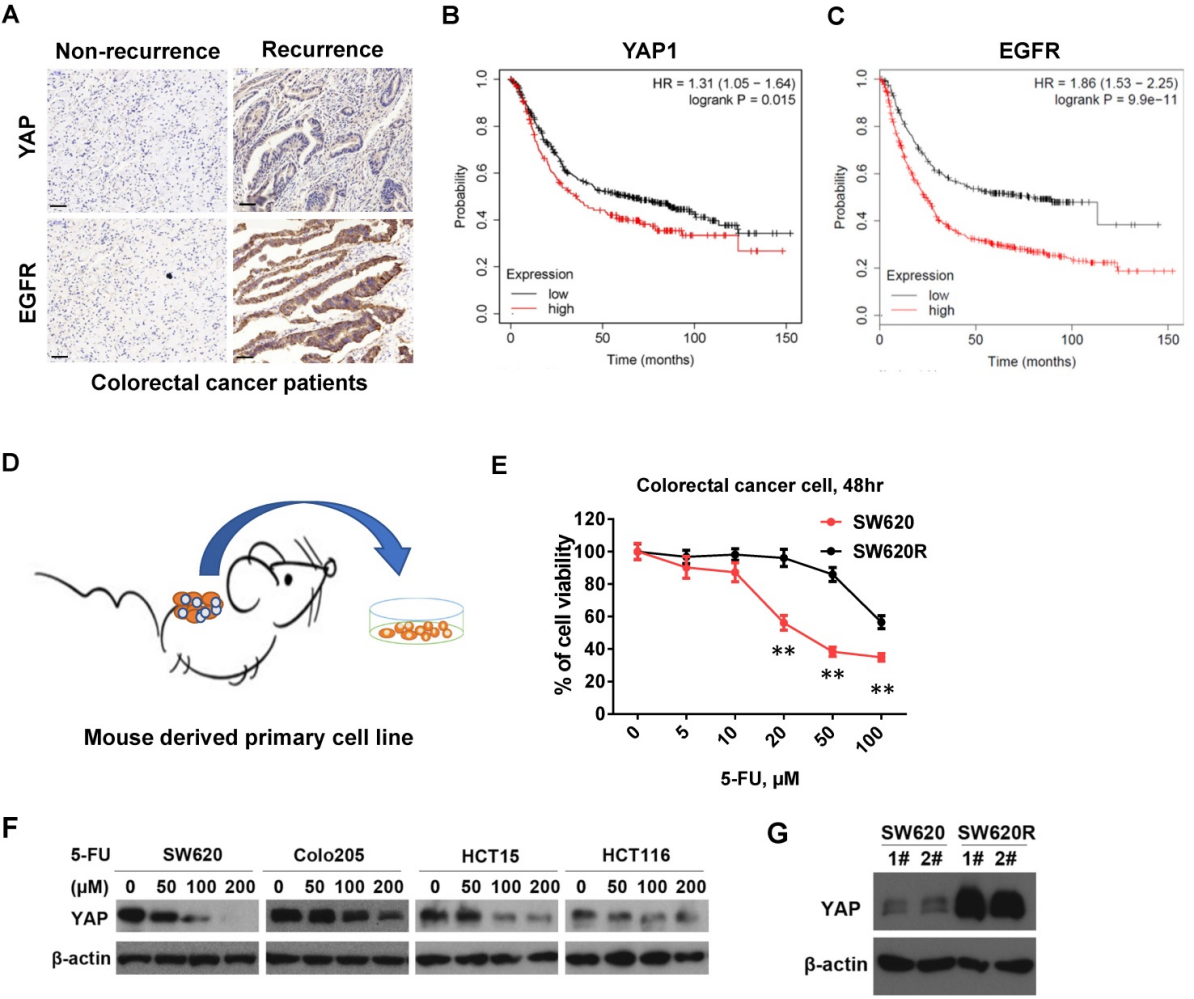
** YAP protein levels by 5-Fu in CRC cells. A.** Immunohistochemical (IHC) analysis of the expression of YAP, EGFR in human CRC tissue sections (200X) (Non-recurrence, recurrence: the CRC patients without or with recurrence in the first year after radical resection). Scale bars represent 50 μm. **B-C.** Kaplan-Meier survival curves comparing overall survival rates on the basis of high and low expression of YAP1 (B) or EGFR (C) in colorectal cancer patient cohort (https://kmplot.com/). **D.** A schematic diagram of primary cell culture of 5-Fu resistant CRC cells derived from NOD/SCID mouse. **E.** MTT cell viability analysis of SW620R and SW620 cells treated with various concentration of 5-Fu for 48 hr. **F.** Western blot analysis of YAP expression in various colorectal cancer cells treated with various concentration of 5-Fu for 48 hr. **G.** Western blot analysis of YAP expression in SW620 and SW620R cells. The result in E represents the mean ± SD. **p < 0.01.

First, we examined the expression of YAP and EGFR in paraffin-embedded sections of 84 human CRC tissue samples (36 samples of CRC non-recurrence, 48 samples of CRC recurrence) by immunohistochemistry analysis. We found that staining intensity of both YAP and EGFR in CRC recurrence was much stronger than those in CRC non-recurrence (Figure 1A and Table 1). In addition, YAP expression was positively correlated with EGFR expression in CRC recurrence (Figure 1A, Table 2, and Table 3). Notably, the patient with high expression of YAP1 or EGFR had a lower survival rate than the patient with low expression of YAP1 or EGFR from Kaplan-Meier survival analysis (Figure 1B and 1C). These results suggest that the expression of YAP and EGFR is manifested in human CRC recurrence and correlated with survival rate of CRC patients.

### 5-Fu resistance increases YAP protein levels in CRC cells

To establish the 5-Fu resistant colorectal cancer (CRC) cell line, we employed mouse chemotherapy resistance model. We injected SW620 cells into the flanks of NOD/SCID mice with 5-Fu (50mg/kg*3/ week) and monitored tumor growth. After four weeks, we collected the tumors and isolated the primary cells (named SW620R) (Figure 1B). To examine 5-Fu resistance of SW620R cells, we tested and compared the cell viability by 5-Fu treatment in both SW620 and SW620R cells. We found that viability of SW620R cells was much higher than that of SW620 cells, suggesting SW620R obtained the resistance to 5-Fu (Figure 1C). We next examined the YAP protein levels in various CRC cells including SW620, Colo205, HCT15, and HCT116 cells by 5-Fu treatment and found that 5-Fu decreased YAP protein levels in dose-dependent manner (Figure 1D). In addition, we examined YAP protein levels in both SW620 and SW620R cells and interestingly found that YAP protein levels in SW620R cells was much higher than those in SW620 cells (Figure 1E). These results suggested that 5-Fu decreased the YAP protein levels while 5-Fu resistance increased YAP protein levels in CRC cells.

### YAP promotes tumorigenicity of CRC cells

**Figure 2:**
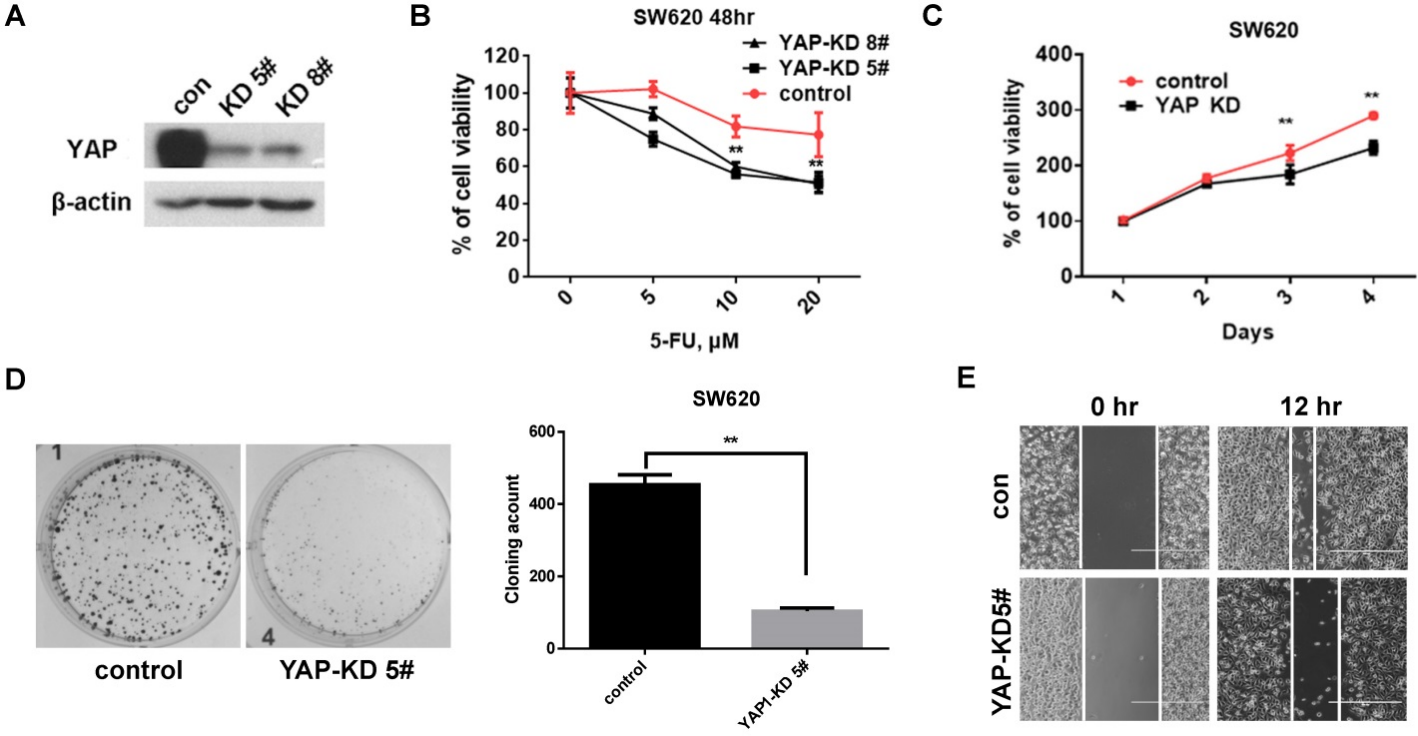
** YAP increased CRC cell proliferation, colony formation, and migration. A.** Western blot analysis of YAP expression in SW620 cells engineered to stably express control shRNA (Control) and YAP shRNA (YAP-KD #5, #8). **B.** MTT cell viability analysis of control or YAP-KD SW620 cells (YAP-KD #5, #8) treated with various concentration of 5-Fu for 48 hr. **C.** MTT cell proliferation rates analysis of control or YAP-KD SW620 cells (YAP-KD #5) for 4 days. **D.** Colony formation assay of control or YAP-KD SW620 cells (YAP-KD #5). Representative images (left) and quantification of the colonies (right). **E.** Wound healing assay of control or YAP-KD SW620 cells (YAP-KD #5). The results in B and C represent the mean ± SD. **p < 0.01.

To investigate the role of YAP in CRC cells, we established stable YAP knockdown (KD) cell lines (Figure 2A). We first tested the viability by 5-Fu treatment under YAP depletion conditions. The viability of YAP-KD cells was lower than that of control cells by 5-Fu treatment (Figure 2B). We next evaluated tumorigenicity under YAP depletion conditions using MTT assay, colony formation assay, and scratch wound-healing assay. We found that cell proliferation (Figure 2C), colony formation (Figure 2D), and migration (Figure 2E) were significantly decreased in YAP-KD cells compared to control. These results indicated that YAP provided resistance to 5-Fu treatment in CRC cells and promoted *in vitro* tumorigenicity of CRC cells.

### EGFR/YAP signaling drives 5-Fu resistance in CRC cells

**Figure 3:**
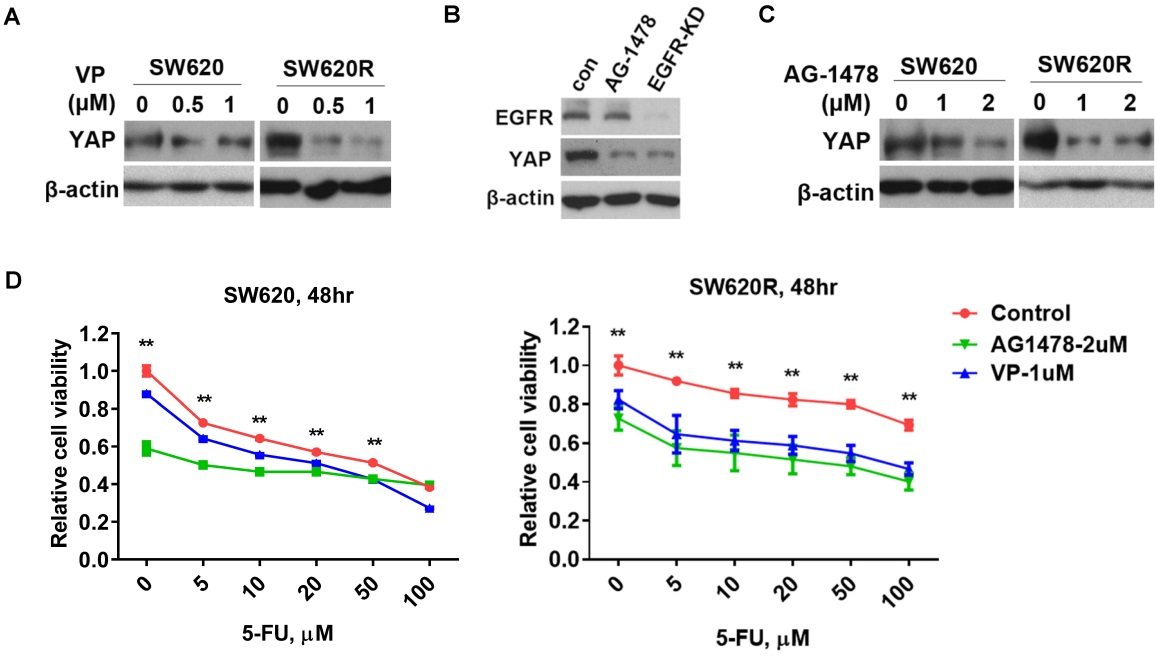
** YAP/EGFR synergistically drive 5-Fu resistance in CRC cells. A.** Western blot analysis of YAP expression in SW620 and SW620R cells treated with various concentration of VP for 48 hr. **B.** Western blot analysis of YAP expression in SW620 cells treated with AG1478 (2 μM) or infected with EGFR shRNA (EGFR-KD) for 48 hr. **C.** Western blot analysis of YAP expression in SW620 cells treated with various concentration of AG1478 for 48 hr. **D.** MTT cell viability analysis of SW620 (Left) or SW620R (Right) cells treated with combination of VP (1 μM) or AG1478 (2 μM) and various concentration 5-Fu for 48 hr. The result in D represents the mean ± SD. **p < 0.01.

Since YAP increased tumorigenicity of CRC cells (Figure 2D and 2E), we next investigated whether YAP regulated 5-Fu resistance in CRC cells. We examined YAP protein levels by treatment of verteporfin (VP), an inhibitor targeting YAP interaction with TEAD, in CRC cells. We found that VP significantly decreased YAP protein levels in SW620R cells, but not in SW620 cells (Figure 3A). Since EGFR signaling pathway has crosstalk with the Hippo/YAP signaling pathway in various cancers including CRC 13, we further investigated whether EGFR regulated YAP protein levels in CRC cells. We tested YAP protein levels by either treatment of AG1478, one of EGFR inhibitors, or stable knockdown of EGFR. EGFR inhibition or knockdown decreased YAP protein levels in SW620 cells (Figure 3B), suggesting EGFR positively regulated YAP protein levels. To assess the susceptibility to EGFR inhibition in 5-Fu resistant cells, we examined YAP protein levels by treatment of AG1478. EGFR inhibition more significantly decreased YAP protein levels in SW620R cells than those in SW620 cells (Figure 3C). To further confirm the role of YAP and EGFR in 5-Fu resistant cells, we examined the cell viability using VP and AG1478. The viability of SW620R cells was more significantly decreased than that of SW620 cells by treatment of VP and AG1478 in the presence of 5-Fu (Figure 3D). These results suggested that EGFR/YAP signaling drove the 5-Fu resistance of CRC cells.

### Combinational inhibition of YAP and EGFR suppressed 5-Fu resistance *in vitro* and *in vivo* in CRC

**Figure 4:**
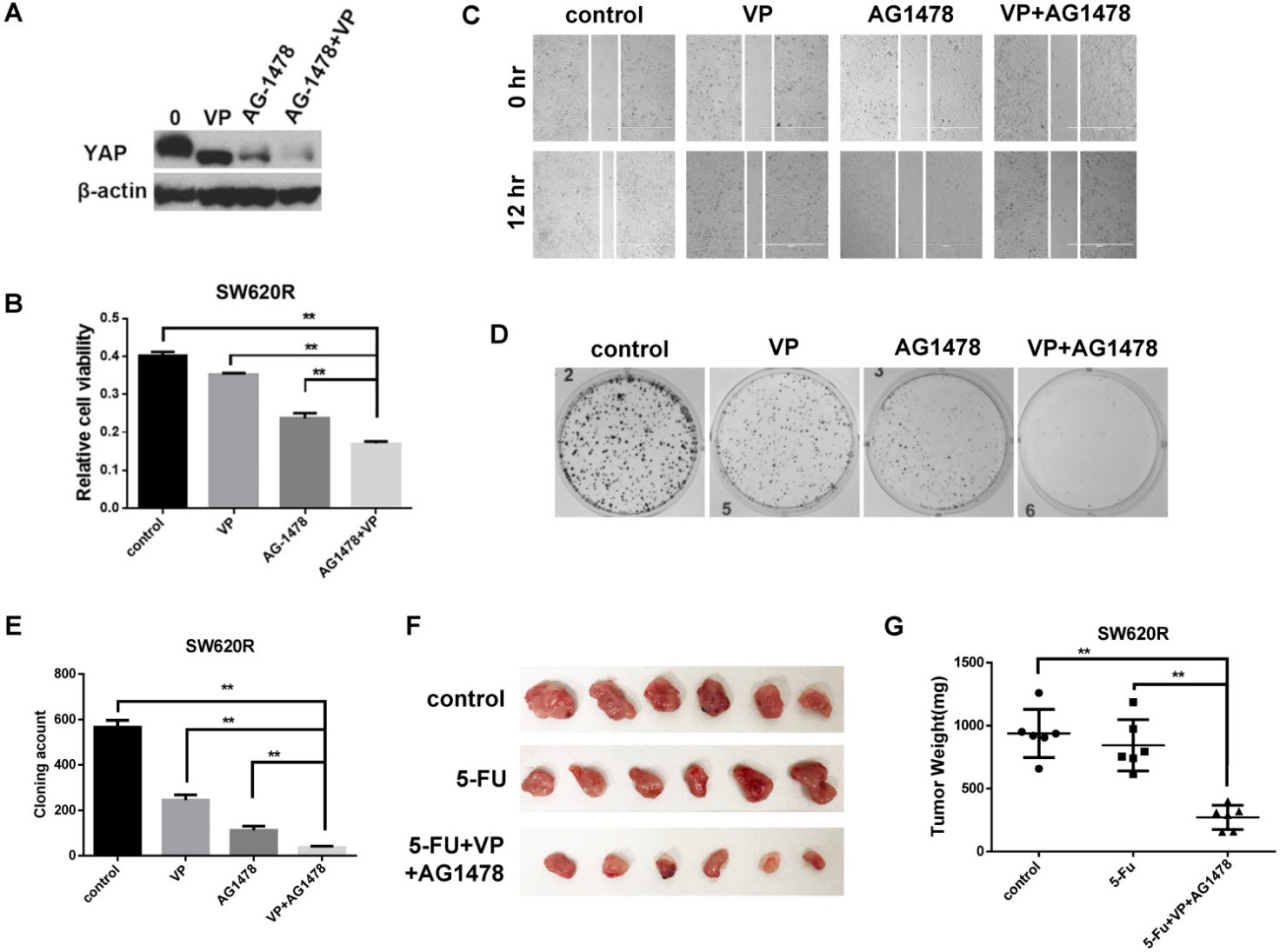
** Combinational inhibition of YAP and EGFR synergistically suppresses 5-Fu resistance.** A. Western blot analysis of YAP expression in SW620R cells treated with VP (1 μM), AG1478 (2 μM), or combination of VP and AG1478 (VP+AG1478) for 48 hr. B. MTT cell viability analysis of SW620R cells treated with VP (1 μM), AG1478 (2 μM), or combination of VP and AG1478 (VP+AG1478) for 48 hr. C. Wound healing assay of SW620R cells treated with VP (1 μM), AG1478 (2 μM), or combination of VP and AG1478 (VP+AG1478) for 12 hr. D, E. Colony formation assay of SW620R cells treated with VP (1 μM), AG1478 (2 μM), or combination of VP and AG1478 (VP+AG1478). Representative images (D) and quantification of the colonies (E). F, G. Xenograft tumor development in NOD/SCID mice inoculated with SW620R cells treated with vehicle (control), 5-FU (30mg/kg*3/ week, i.p.), or the combination of VP+AG1478 with 5-FU (VP 50mg/kg*3/ week, AG1478 50mg/kg*3/ week, 5-FU 30mg/kg*3/ week, i.p.) for 2 weeks. After 2 weeks, mice were sacrificed, and tumors were collected (F) and weighted (G). (n=6) The results in B, E, and G represent the mean ± SD. **p < 0.01.

Since YAP and EGFR regulated 5-Fu resistance in CRC cells (Figure 3D), we next investigated whether combinational inhibition of YAP and EGFR could synergistically reduce chemotherapy resistance. We first examined the YAP protein levels by combinational treatment of VP and AG1478. The combinational treatment synergistically reduced the YAP protein levels compared with single treatment of VP or AG1478 in SW620R cells (Figure 4A). We next examined *in vitro* tumorigenicity by combinational treatment of VP and AG1478 in SW620R cells using MTT, scratch wound-healing assay, and colony formation assay. The combinational treatment significantly decreased cell viability (Figure 4B), migration (Figure 4C), and colony formation (Figure 4D and 4E) in SW620R cells compared to the control. To further confirm the effect of combinational treatment, we tested *in vivo* tumorigenicity using mouse xenograft model. We compared the therapeutic effects between single treatment of 5-Fu and combinational treatment of 5-Fu, VP, and AG1478 for SW620R xenograft tumors. The combinational treatment of 5-Fu, VP, and AG1478 more effectively suppressed the tumor growth whereas single treatment of 5-Fu had no significant difference on the tumor growth compared to the control (Figure 4F and 4G). These results suggested that the combinational treatment of VP and AG1478 significantly reduced 5-Fu resistance *in vivo* and *in vitro* in CRC.

## Discussion

CRC is characteristic of poor prognosis and high death rate, as the 3rd most common cancer in pan cancer statistics worldwide 14. It has been reported that the expression of YAP is associated with the cancer cell proliferation and chemotherapy resistance in solid tumors 11, 15-17. YAP, a transcriptional coactivator in the Hippo signaling pathway, is bound with TEAD via translocating into the cell nucleus 18. In this study, we found that YAP expression of CRC recurrence was correlated with EGFR expression, which were manifested in human CRC patient samples (Figure 1A, and Table 1 and 2). In addition, initial treatment of 5-Fu led to reduction of YAP protein levels while 5-Fu resistance highly increased YAP protein levels in CRC (Figure 1). We also found that YAP played important role in cell proliferation, migration, and 5-Fu resistance in CRC cells (Figure 2). These indicated that YAP is as well associated with cancer development and chemotherapy resistance in CRC recurrence.

VP (an inhibitor of YAP) reduces the translocation and expression of YAP by specially blocking YAP-TEAD complex 19. Since single inhibition of YAP by VP could not efficiently inhibit cell viability of 5-Fu resistant CRC cells, we considered another therapeutic target related with YAP signaling. EGFR signaling pathway is related with YAP signaling pathway 20, 21, and AG1478 (an inhibitor of EGFR/ErbB1) induces the phosphorylation and degradation of YAP in plasma 11, 21. We showed that YAP expression of CRC recurrence was correlated with EGFR expression in human CRC patient samples (Figure 1A, and Table 1 and 2), suggesting EGFR is related with YAP in CRC recurrence. We next examined whether inhibition of EGFR could suppress YAP protein levels by AG1478 treatment or EGFR knockdown in CRC. Interestingly, we found that inhibition of EGFR induced decrease of YAP protein levels by either AG1478 treatment or EGFR knockdown. We next examined the synergistic effect on the inhibition of YAP protein levels by combinational treatment of VP and AG1478 in CRC. We found the combinational inhibition of YAP and EGFR synergistically suppressed cell viability, colony formation, and migration in SW620R cells (Figure 4A-4E). In addition, the combinational treatment of 5-Fu with VP and AG1478 reversed the chemotherapy resistance in mouse xenograft model of CRC (Figure 4F and 4G). These indicated that EGFR is related with chemotherapy resistance, which could be a therapeutic target for chemotherapy resistant CRC.

5-Fu is one of the initial chemotherapeutic drugs for colorectal cancer patients 22, 23. Because primary or secondary resistance to 5-Fu make treatment failure, metastatic CRC patients maintain a low 5-year-survival rate. Therefore, resistance to 5-FU has been a major obstacle in chemotherapy for advanced CRC patients. It has been reported several targets that inhibit 5-Fu resistance in CRC. For instance, 5-Fu resistance in CRC was more affected by cytoplasmic localization of expressed Nrf2 (cNrf2) than by nuclear localization (nNrf2) 24, 25. RV-59, a nitrogen-substituted anthra[1,2-c]^1^,^2^,^5^ thiadiazole-6,11-dione derivative, was suggested as anti-tumor agent which effectively suppressed cNrf2, which reversed chemotherapy resistance in CRC 26. Andrographolide synergizes the cytotoxic effects of 5-FU in CRC by targeting BAX, which provides combinational treatment strategies for chemotherapy resistance in CRC patients with expressing low level of BAX protein 27. Here, we suggested EGFR as therapeutic target of CRC treatment, which synergized effect of YAP inhibition by VP in 5-Fu resistant CRC.

In the present study, we confirmed the role of YAP in CRC tumorigenicity *in vitro* and *in vivo*. We also found that the regulation of YAP by 5-Fu is one of the major mechanisms underlying YAP-driven CRC development and chemotherapy resistance. Furthermore, our results suggested that the combinational inhibition of EGFR and YAP provided synergistic efficacy of chemotherapy resistant CRC treatment. In conclusion, our study will not only enhance our understanding of the EGFR/YAP signaling pathway in chemotherapy resistant CRC development and progression, but it will also provide a new strategy for CRC treatment in chemotherapy resistance.

## Figures and Tables

**Table 1:** Correlation between the staining of YAP and EGFR (Chi-square test)

	n	YAP				EGFR		
		+	-	*P*		+	-	*P*
Non-recurrence	36	13	23	<0.05		27	9	<0.05
Recurrence	48	32	16			36	12	

**Table 2:** Correlation between the staining of YAP and EGFR (Pearson test)

		EGFR			
		-	+	r	*P*
Non-recurrence	YAP				
	-	6	7	0.367	0.028
	+	3	20		
Recurrence	YAP				
	-	12	4	0.816	0.000
	+	0	32		

**Table 3:** Correlation between the staining of YAP, EGFR and clinicopathologic characteristics in 84 cases of human colorectal cancer tissues

	n	YAP				EGFR		
		-	+	*P*		-	+	*P*
**Age(years)**								
≤60	75	26	49	0.999		25	55	0.431
>60	9	3	6			1	8	
**Gender**								
**Male**	48	20	28	0.163		15	33	0.202
**Female**	36	9	27			6	30	
**Depth of tumor invasion**								
T1-T2	31	17	14	0.004		13	18	0.009
T3-T4	53	12	41			8	45	
**Histologic type**								
Poor and undifferentiated	34	10	24	0.487		9	25	0.803
Well and moderate	50	19	31			12	38	
**Metastasis**								
No	33	18	15	0.002		13	20	0.020
Yes	51	11	40			8	43	
